# Author Correction: Reproductive phasiRNAs regulate reprogramming of gene expression and meiotic progression in rice

**DOI:** 10.1038/s41467-023-37355-6

**Published:** 2023-03-22

**Authors:** Yu-Chan Zhang, Meng-Qi Lei, Yan-Fei Zhou, Yu-Wei Yang, Jian-Ping Lian, Yang Yu, Yan-Zhao Feng, Ke-Ren Zhou, Rui-Rui He, Huang He, Zhi Zhang, Jian-Hua Yang, Yue-Qin Chen

**Affiliations:** 1 Guangdong Provincial Key Laboratory of Plant Resources, State Key Laboratory for Biocontrol, School of Life Science, Sun Yat-Sen University, Guangzhou, 510275 P. R. China; 2 Guangdong Laboratory of Lingnan Modern Agriculture, Guangzhou, 510642 China

**Keywords:** Plant sciences, Pollen

Correction to: *Nature Communications* 10.1038/s41467-020-19922-3, published online 27 November 2020

In this article the author name Ke-Ren Zhou was incorrectly written as Ke-Reng Zhou. The original article has been corrected.

