# Supplementary material for: Reproductive phasiRNAs regulate reprogramming of gene expression and meiotic progression in rice
Source: Nat Commun. 2020 Nov 27;11:6031. doi: 10.1038/s41467-020-19922-3 (PMC7695705; doi:10.1038/s41467-020-19922-3)
Supplement: Supplementary file 1 — Supplementary Information [file 41467_2020_19922_MOESM1_ESM.docx]

Supplementary Information

**Reproductive phasiRNAs regulate reprogramming of** **gene expression and meiotic progression in rice**

**Zhang et al.**


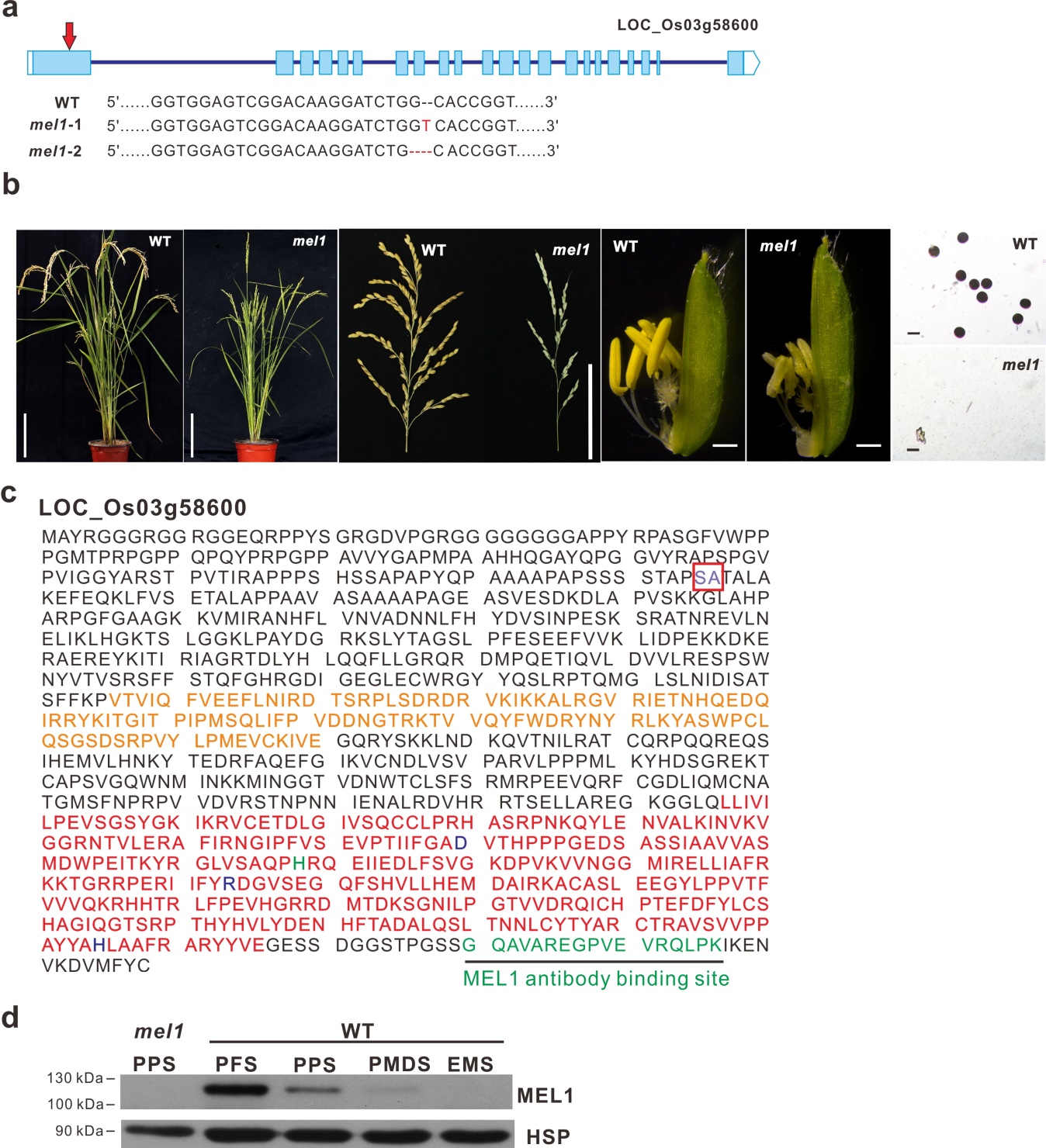


Supplementary Figure 1. Gene structure of *MEL1*, and phenotype and immunoblot of MEL1 protein in *mel1* and wild-type (WT) plants. a Schematic of *mel1* knockout mutants generated by CRISPR-Cas9. The red arrowhead indicates the gRNA target site. The genotypes of *mel1* mutants used in this study were showed below the schematic. b Gross morphology, panicles, spikelets and pollen grains of wild-type and *mel1* plants. Scale bars, 15 cm for plants, 10 cm for panicles, 2 mm for spikelets and 50 µm for pollen grains. n = 3 independent replicates. c Gene structure of *MEL1*. The red box indicates the gRNA target site by CRISPR/Cas9. The green letters indicate the conserved histidine residue, the blue letters indicate the DDH residues, the orange letters indicate the PAZ domain, and the red letters indicate the PIWI domain. The MEL1 antibody binding site was also showed in green color. d Immunoblot of the MEL1 protein in *mel1* and wild-type plants during anther development. n = 3 independent replicates. Antibody for MEL1 was produced by using oligopeptides that were previously reported [^1^](#_ENREF_1).


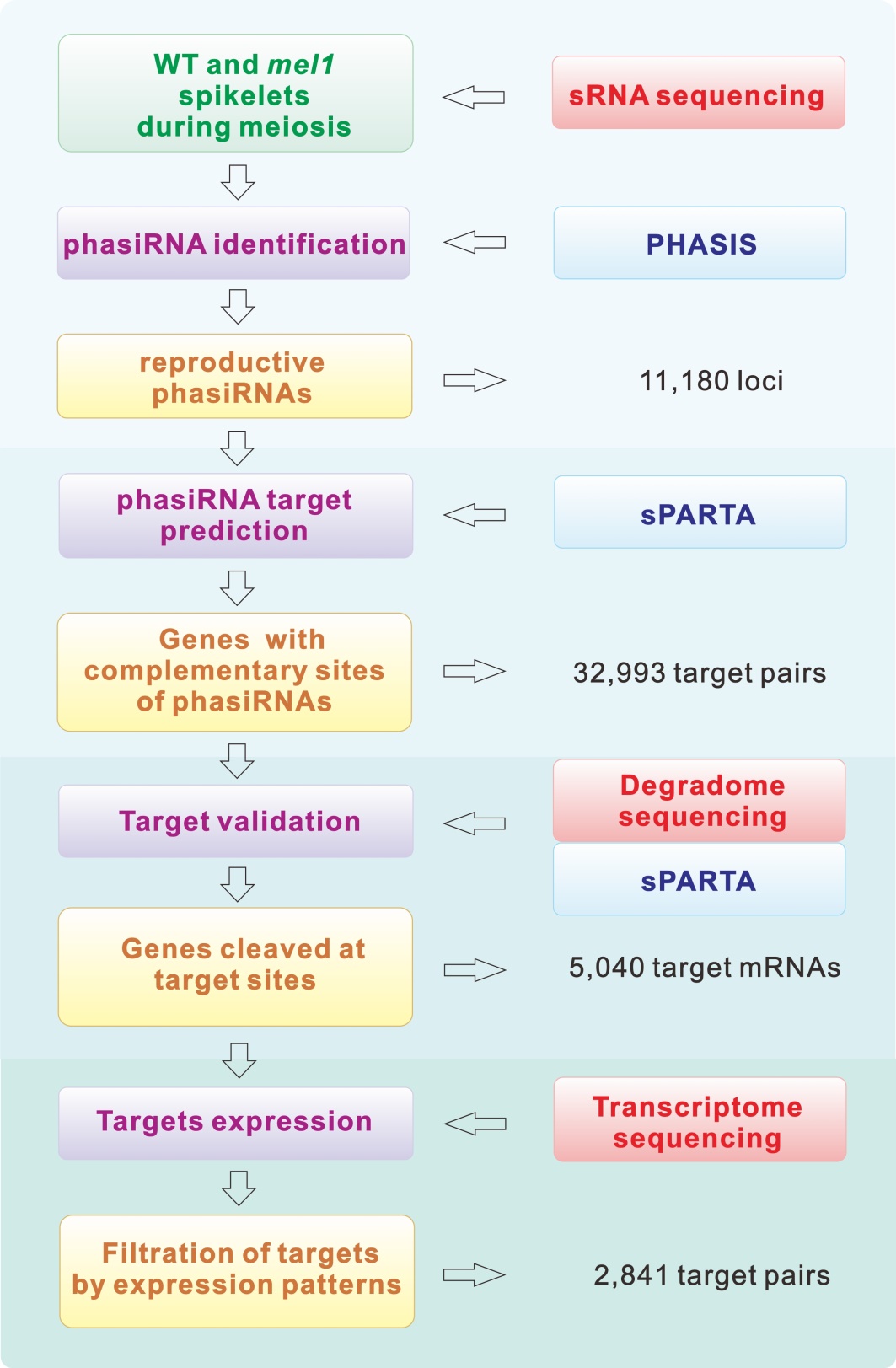


Supplementary Figure 2. An integrative strategy and pipeline for the systematic identification of reproductive phasiRNAs and their target genes during meiosis in rice. Spikelets of WT PMC Formation Stage (PFS), PMC Prophase Stage (PPS), PMC Meiotic Divisions Stage (PMDS), and Early Microspore Stage (EMS) and *mel1* spikelets at PPS were collected for sRNA sequencing, degradome sequencing and transcriptome sequencing.


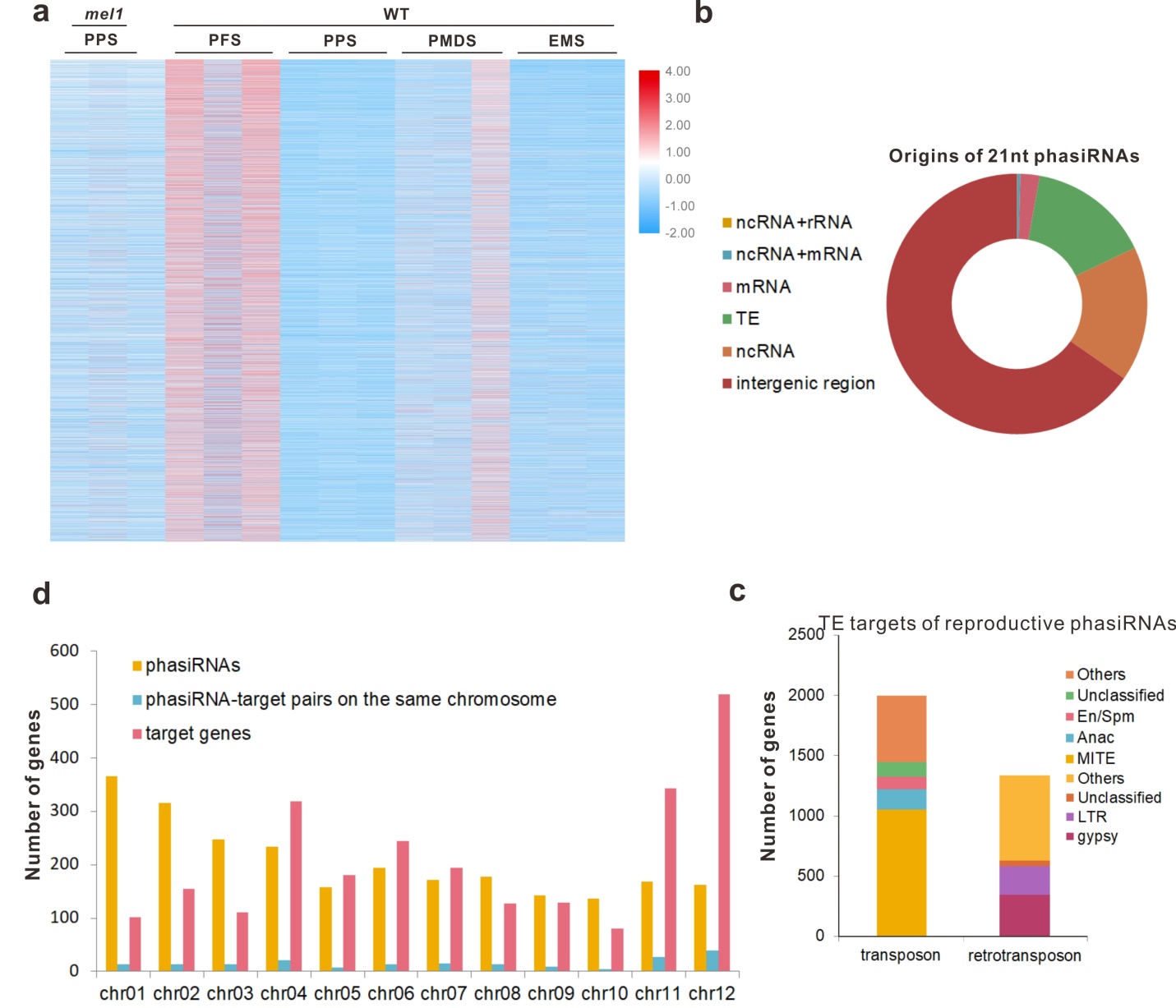


Supplementary Figure 3. Analysis of reproductive phasiRNAs and their targets. a Heatmap of reproductive phasiRNAs in WT and *mel1* spikelet at PFS, PPS, PMDS and EMS. PhasiRNA expression values used to create the heatmap are the log2 RPM of phasiRNAs, and the values were scaled by row. b The origins of 21-nt phasiRNAs. The data were generated from 2,350 *PHAS* loci. c The categories of TE targets of phasiRNAs. The data were generated from 3,340 TE target genes of phasiRNAs. d The distribution of phasiRNAs and their target genes on different chromosomes. The data were generated from 2,474 phasiRNAs and 2,505 target genes.


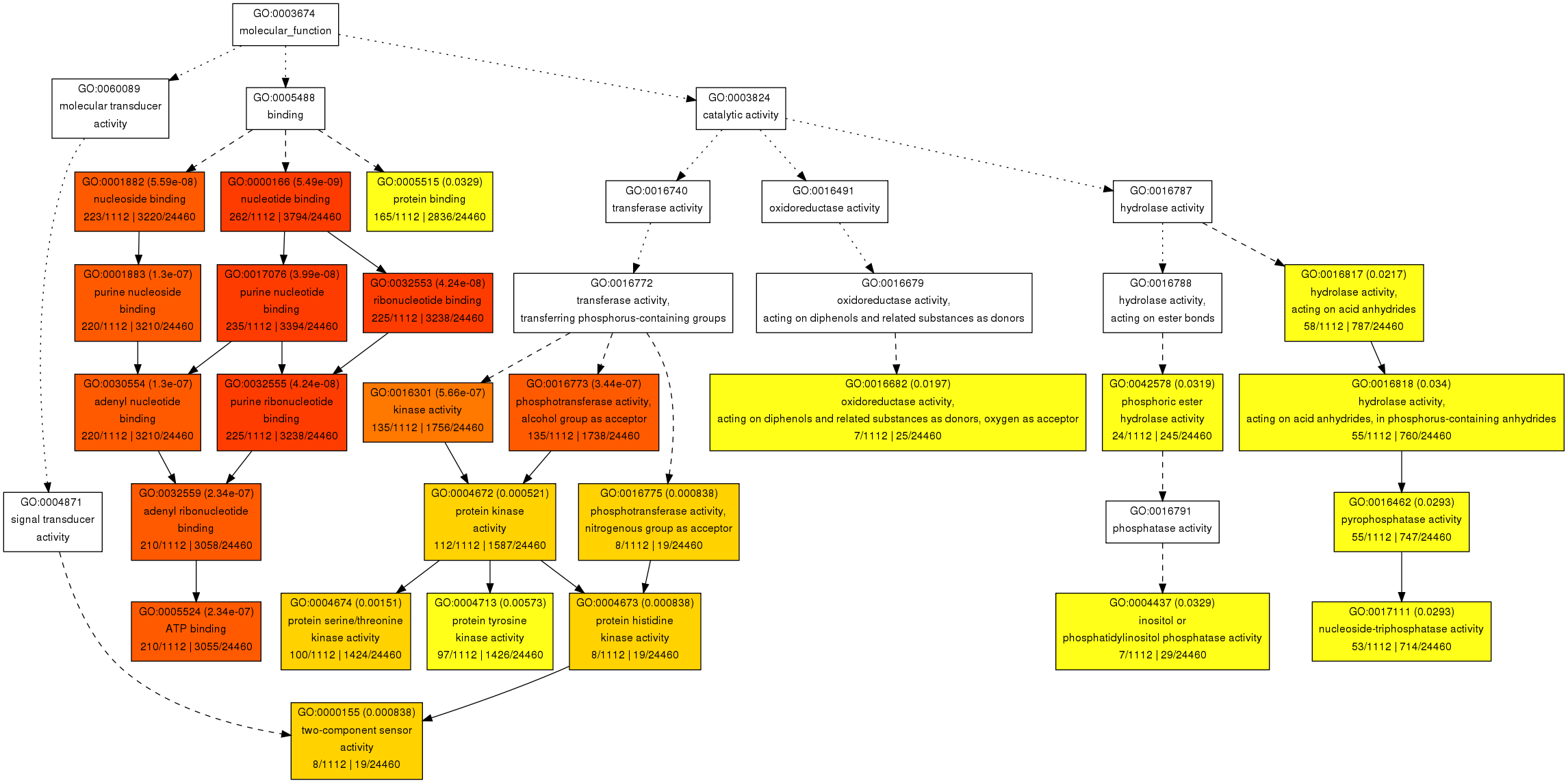


**Supplementary Figure 4**. **GO analysis of target mRNAs of reproductive phasiRNAs.**


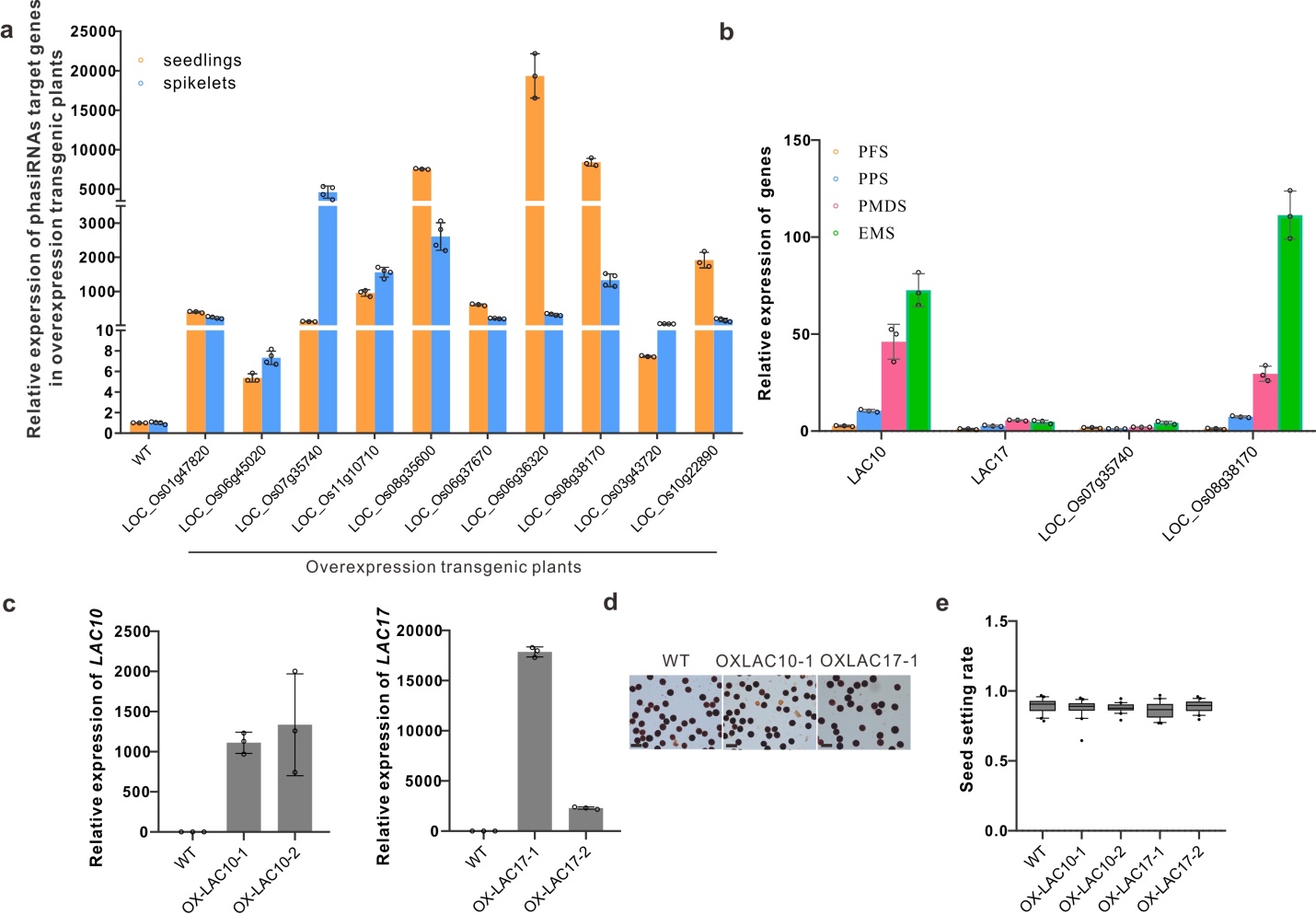


**Supplementary Figure 5**. **Expression and phenotype analysis of phasiRNA** **target genes and control genes.** **a** Relative expression of phasiRNA target genes in spikelets and seedlings of overexpression transgenic plants. Values are means ± SD (*n* = 3 replicates for seedlings and *n* = 4 replicates for spikelets). **b** Relative expression of two control genes (*LAC10* and *LAC17*) and two phasiRNA target genes in WT spikelets at PFS, PPS, PMDS and EMS. Values are means ± SD (*n* = 3 replicates). **c** Relative expression level of control genes in their overexpression transgenic plants. Values are means ± SD (*n* = 3 replicates). (a-c) normalized against *Actin2*. **d** The pollen grains of control transgenic plants stained with iodine-potassium iodide. Scale bars = 100 µm. n = 3 independent replicates. **e** The seed setting rate of WT and control transgenic plants. Box plots indicate median (middle line), 25th, 75th percentile (box) and 10th and 90th percentile (whiskers) as well as outliers (single points). n = 20 plants


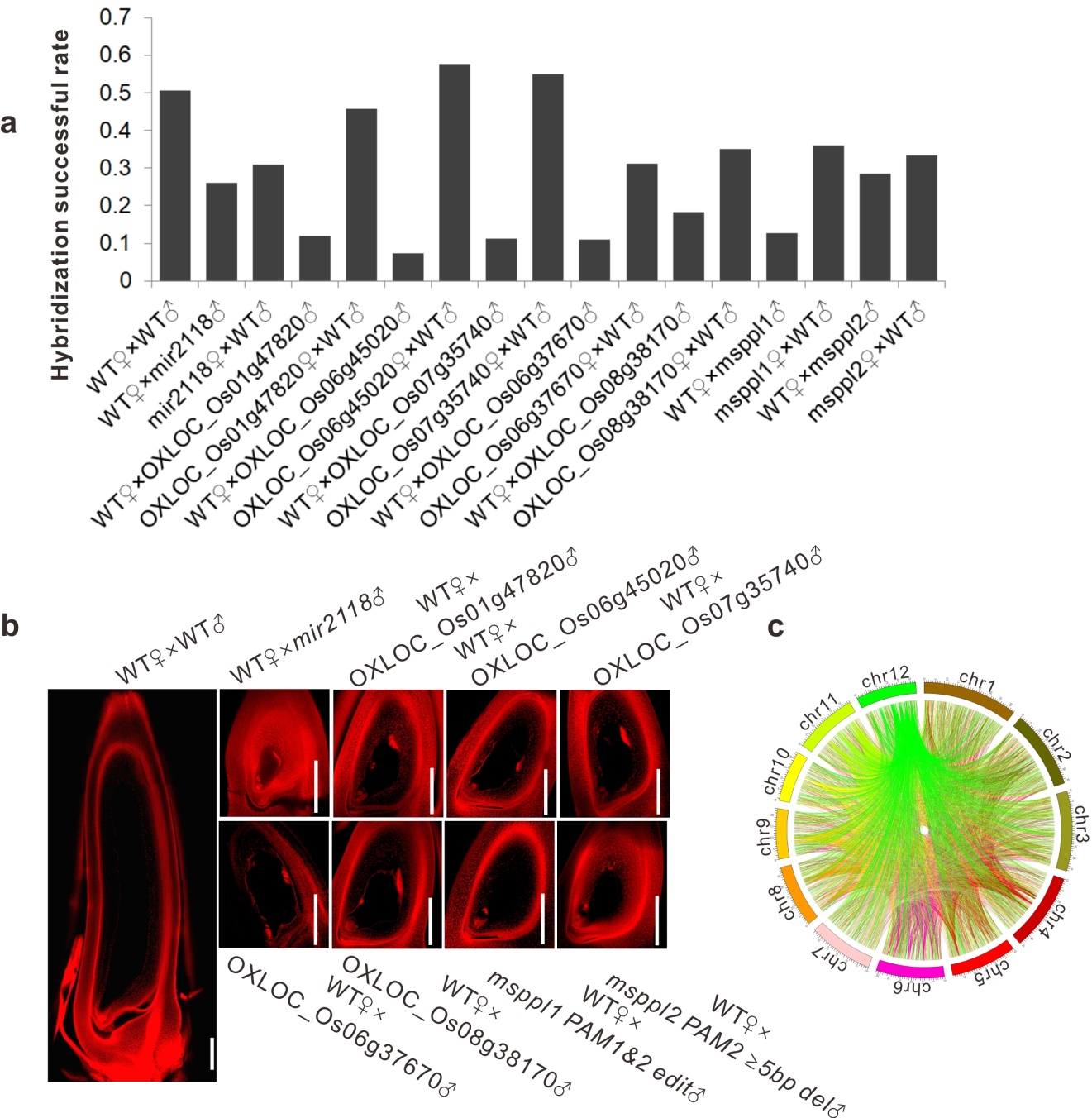


**Supplementary Figure 6**. **Hybridization between mutants and WT plants.** **a** The hybridization successful rate of different group. n = 336, 249, 55, 133, 46, 148, 26, 62, 20, 81, 167, 77, 97, 55, 125, 155 and 51 spikelets from left to right respectively. **b** The Ovary of hybrids 3 days after cross. Scale bars, 200 µm. n = 3 independent replicates. **c** The targeting links between phasiRNAs that have more than two targets and targets on different chromosomes. The color of the linking lines is the same as that of the chromosome from which the phasiRNA is derived.


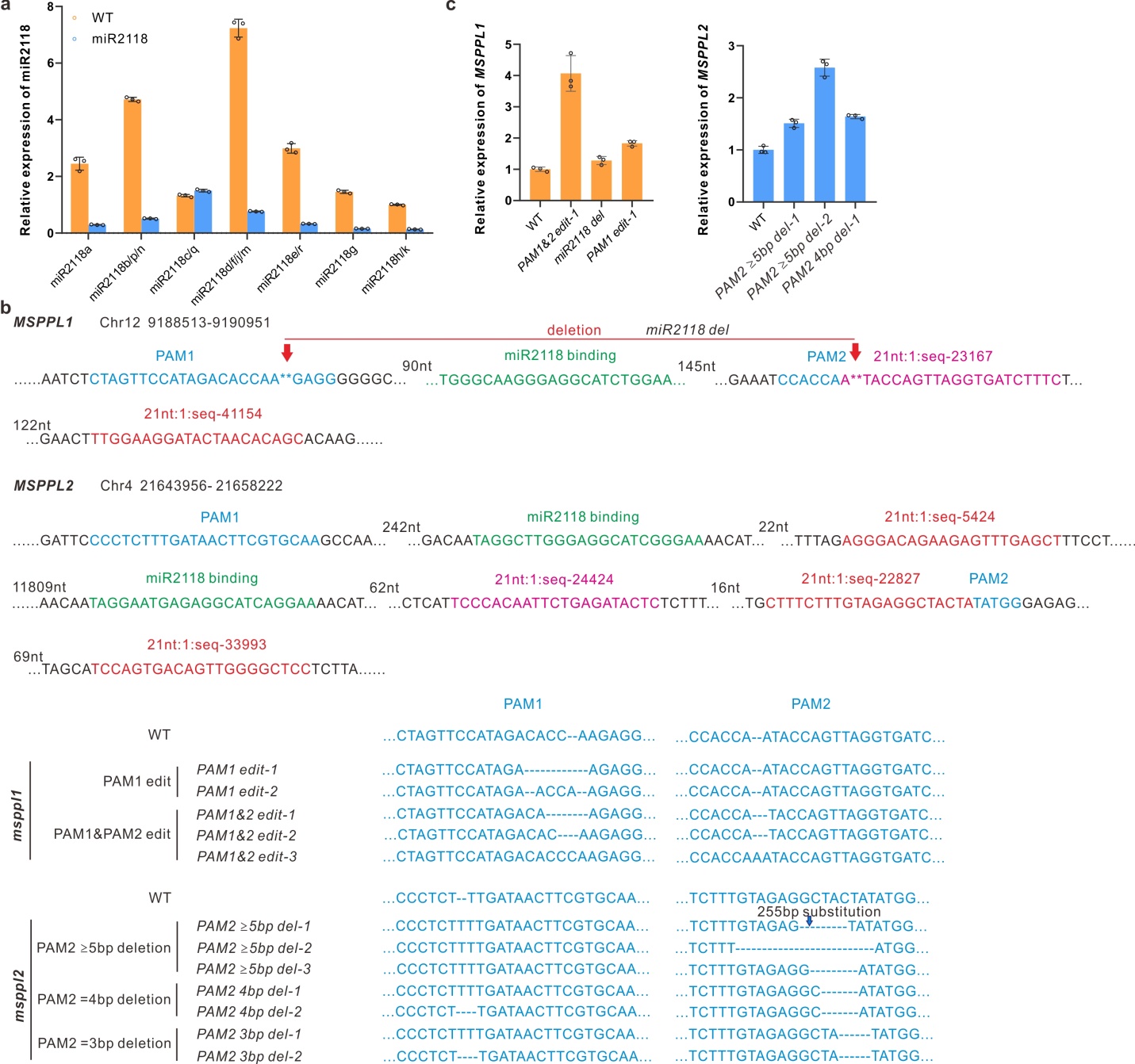


**Supplementary Figure 7**. **Expression of miR2118, *MSPPL1* and *MSPPL2* in WT and mutants and the information of *MSPPL1*, *MSPPL2* and different mutants.** **a** Relative expression of miR2118 in WT and *mir2118* panicles. The mir2118 plants were generated by Short Tandem Target Mimic Technology (STTM) which knockdown most of the miR2118 family members with similar sequences. Values are means ± SD (n = 3 replicates), normalized against *U6*. **b** The gene structure of *MSPPL1* and *MSPPL2* and the genotypes of different mutants used in this study. phasiRNAs (sense sequence) were marked in red, phasiRNAs (antisense sequence) were marked in purple, miR2118 binding sites were marked in green and the edited regions in mutants were marked in blue. **c** Relative expression of *MSPPL1* and *MSPPL2* in the wild type (WT) and different mutants at the PFS. Values are means ± SD (n = 3 replicates, normalized against *Actin2*).


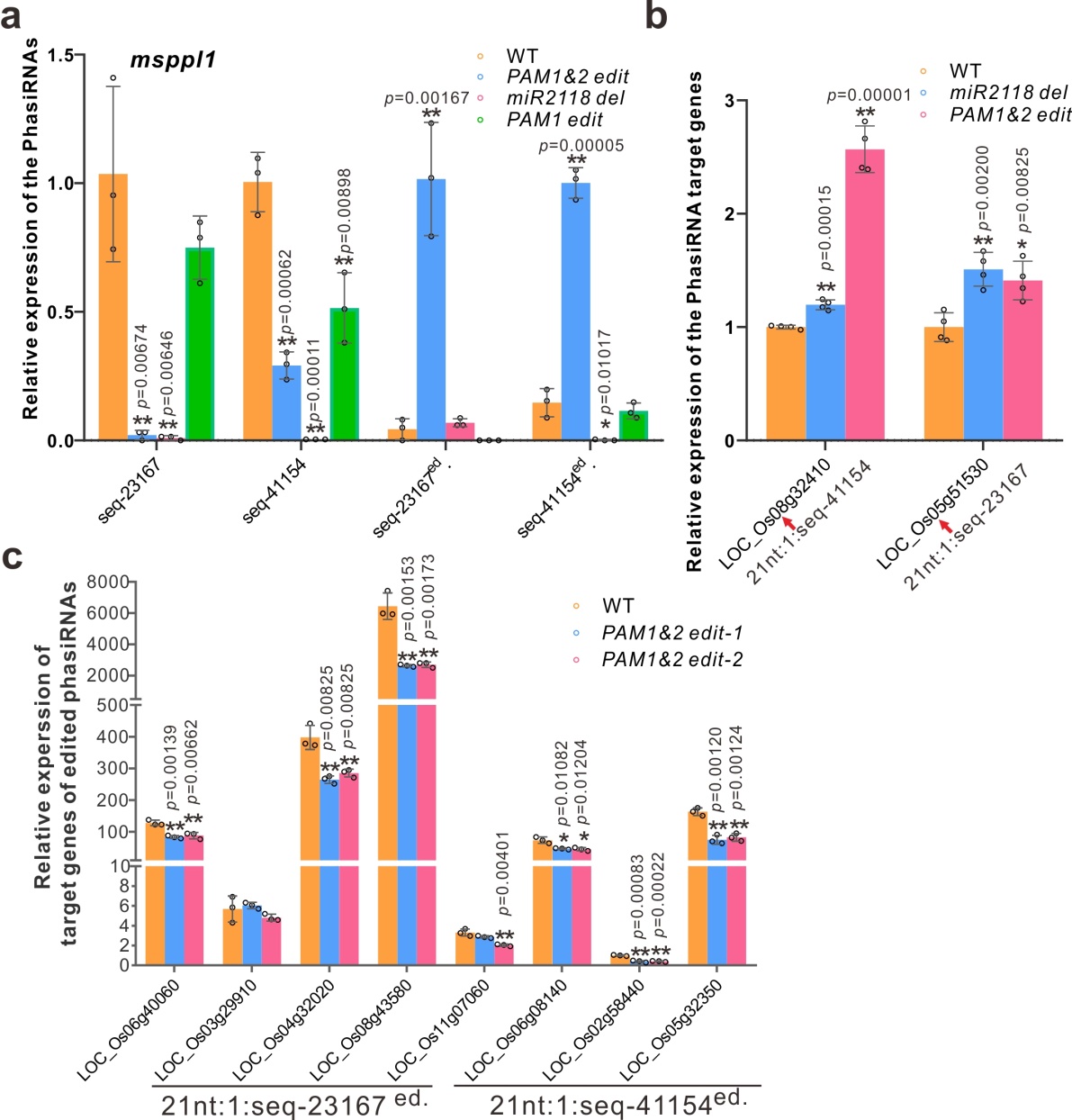


**Supplementary Figure 8**. **Expression analysis of the phasiRNAs and their targets.** **a** Relative expression of phasiRNAs generated from *MSPPL1* and phasiRNAs^ed.^ generated from *msppl1* mutants harboring the edited sequence. The values are the means ± SD (n = 3 replicates, normalized against *U6*). **b** Relative expression of target genes of phasiRNAs generated from *MSPPL1* in the *msppl1* mutant. The values are the means ± SD (n = 4 replicates, normalized against *Actin2*). **c** Relative expression of target genes of edited phasiRNAs (ed.) in the *msppl1* mutant. The values are the means ± SD (n = 3 replicates, normalized against *Actin2*). Significant differences were identified at the 5% (*) and 1% (**) probability levels using two-tailed paired *t*-test.

Supplementary references

1. Komiya, R. et al. Rice germline-specific Argonaute MEL1 protein binds to phasiRNAs generated from more than 700 lincRNAs. *Plant J* **78**, 385-97 (2014).
