# Supplementary material for: Reproductive phasiRNAs regulate reprogramming of gene expression and meiotic progression in rice
Source: Nat Commun. 2020 Nov 27;11:6031. doi: 10.1038/s41467-020-19922-3 (PMC7695705; doi:10.1038/s41467-020-19922-3)
Supplement: Supplementary file 3 — Descriptions of Additional Supplementary Files [file 41467_2020_19922_MOESM3_ESM.pdf]

Descriptions of Additional Supplementary Files

Supplementary Data 1

Description: A list of the sequencing data performed in this study.

Supplementary Data 2

Description: Information of reproductive phasiRNAs.

Supplementary Data 3

Description: Information of the target genes of reproductive phasiRNAs.

Supplementary Data 4

Description: Information of other target genes which were filtered out.

Supplementary Data 5

Description: Enriched GO terms of reproductive phasiRNA targeted genes.

Supplementary Data 6

Description: Primers used in this study.
