# Supplementary material for: Reproductive phasiRNAs regulate reprogramming of gene expression and meiotic progression in rice
Source: Nat Commun. 2020 Nov 27;11:6031. doi: 10.1038/s41467-020-19922-3 (PMC7695705; doi:10.1038/s41467-020-19922-3)
Supplement: Supplementary file 10 — Reporting Summary [file 41467_2020_19922_MOESM10_ESM.pdf]

nature research

Corresponding author(s): Yue-Qin Chen, Yu-Chan Zhang

Last updated by author(s): Oct 29, 2020

Reporting Summary

Nature Research wishes to improve the reproducibility of the work that we publish. This form provides structure for consistency and transparency in reporting. For further information on Nature Research policies, see Authors & Referees and the Editorial Policy Checklist .

Statistics

For all statistical analyses, confirm that the following items are present in the figure legend, table legend, main text, or Methods section.

n/a	Confirmed	
☐	☒	The exact sample size (*n*) for each experimental group/condition, given as a discrete number and unit of measurement
☐	☒	A statement on whether measurements were taken from distinct samples or whether the same sample was measured repeatedly
☐	☒	The statistical test(s) used AND whether they are one- or two-sided<br>*Only common tests should be described solely by name; describe more complex techniques in the Methods section.*
☒	☐	A description of all covariates tested
☒	☐	A description of any assumptions or corrections, such as tests of normality and adjustment for multiple comparisons
☐	☒	A full description of the statistical parameters including central tendency (e.g. means) or other basic estimates (e.g. regression coefficient) AND variation (e.g. standard deviation) or associated estimates of uncertainty (e.g. confidence intervals)
☐	☒	For null hypothesis testing, the test statistic (e.g. *F*, *t*, *r*) with confidence intervals, effect sizes, degrees of freedom and *P* value noted<br>*Give P values as exact values whenever suitable.*
☒	☐	For Bayesian analysis, information on the choice of priors and Markov chain Monte Carlo settings
☒	☐	For hierarchical and complex designs, identification of the appropriate level for tests and full reporting of outcomes
☒	☐	Estimates of effect sizes (e.g. Cohen's *d*, Pearson's *r*), indicating how they were calculated

Our web collection on statistics for biologists contains articles on many of the points above.

Software and code

Policy information about availability of computer code

Data collection	Oryza sativa genome assembly RGAP 7.0 was used throughout this study and was downloaded from http://rice.plantbiology.msu.edu/.
Data analysis	Genome-wide searching for PHAS loci and phasiRNA triggers, and phasiRNA expression analysis were performed using the PHASIS package (v3) according to the instructions provided (https://github.com/atulkakrana/PHASIS/wiki). Genome-wide identification of phsiRNA targets was performed using the sPARTA package (v1.26).

For manuscripts utilizing custom algorithms or software that are central to the research but not yet described in published literature, software must be made available to editors/reviewers. We strongly encourage code deposition in a community repository (e.g. GitHub). See the Nature Research guidelines for submitting code & software for further information.

Data

Policy information about availability of data

All manuscripts must include a data availability statement. This statement should provide the following information, where applicable:

- Accession codes, unique identifiers, or web links for publicly available datasets
- A list of figures that have associated raw data
- A description of any restrictions on data availability

The source data for Figs. 1–5 and Supplementary Figs.1, 3, 5–8 are provided as a Source Data file. The sRNA-Seq, degradome and transcriptome datasets were uploaded to the NCBI SRA database (SRA Accession No. PRJNA627552 [https://www.ncbi.nlm.nih.gov/bioproject/PRJNA627552/]). The MEL1 RIP-data was obtained from publicly available sources (DDBJ: DRP000161 [https://ddbj.nig.ac.jp/DRASearch/study?acc=DRP000161]).

Field-specific reporting

Please select the one below that is the best fit for your research. If you are not sure, read the appropriate sections before making your selection.

- [x] Life sciences
- [ ] Behavioural & social sciences
- [ ] Ecological, evolutionary & environmental sciences

For a reference copy of the document with all sections, see nature.com/documents/nr-reporting-summary-flat.pdf

Life sciences study design

All studies must disclose on these points even when the disclosure is negative.

Sample size	10 or more rice plants are usually sufficient for gene expression level analysis and phenotype analysis to eliminate individual difference. In this study, 15 or more individual plants of each transgenic line were obtained and subjected to statistical analyses. For samples that collected for sequencing, the main panicle of more than 120 individual WT plants and more than 40 individual mel1 plants were collected. Then their spikelet at different development stages were collected and subjected for sRNA-seq, transcriptome and degradome.
Data exclusions	All the samples were chosen randomly that there is no exclusion criteria.
Replication	ALL the gene expression level analysis and phenotype analysis have more than three replicates. For the sequencing data, the sRNA and transcriptome of each samples have three replicates. Detailed sequencing information was listed in data S1. For the degradome data, the cleavage of a specific site in each library have been exhibited in data S3. All attempts at replication were successful.
Randomization	All samples were allocated into experimental groups randomly.
Blinding	The investigators were blinded to group allocation during data collection and analysis.

Reporting for specific materials, systems and methods

We require information from authors about some types of materials, experimental systems and methods used in many studies. Here, indicate whether each material, system or method listed is relevant to your study. If you are not sure if a list item applies to your research, read the appropriate section before selecting a response.

Materials & experimental systems

n/a	Involved in the study	
[ ]	[x]	Antibodies
[x]	[ ]	Eukaryotic cell lines
[x]	[ ]	Palaeontology
[x]	[ ]	Animals and other organisms
[x]	[ ]	Human research participants
[x]	[ ]	Clinical data

Methods

n/a	Involved in the study	
[x]	[ ]	ChIP-seq
[x]	[ ]	Flow cytometry
[x]	[ ]	MRI-based neuroimaging

Antibodies

Antibodies used	Antibody for MEL1 protein<br>Anti-Tubulin (cat# 66031-1-Ig,Proteintech)<br>Anti-RPL7 (ab72550,Abcam)<br>Anti-HSP(cat# MBS853567,Mybiosource)
Validation	Anti-MEL1 (GQAVAREGPVEVRQLPKC) were used to induce antibody production in rabbits, Antibody was evaluated using the proper negative controls (mel1) and confirmed specific reactivity in the manuscript.<br>The biological source of Anti-tubulin (cat# 66031-1-Ig,Proteintech) , Anti-RPL7 (ab72550,Abcam) and Anti-HSP(cat# MBS853567,Mybiosource) is mouse ,rabbits and mouse,respectively. Antibodies were validated by the manufacturers and in publications lists one the manufacturers websites.
